# Draft Genome Sequence of CO33, a Coffee-Infecting Isolate of *Xylella fastidiosa*

**DOI:** 10.1128/genomeA.01472-15

**Published:** 2015-12-17

**Authors:** Annalisa Giampetruzzi, Giuliana Loconsole, Donato Boscia, Alessandra Calzolari, Michela Chiumenti, Giovanni P. Martelli, Pasquale Saldarelli, Rodrigo P. P. Almeida, Maria Saponari

**Affiliations:** a Department of Soil, Plant and Food Sciences, University of Bari Aldo Moro, Bari, Italy; b Institute for Sustainable Plant Protection, National Research Council (CNR), Bari, Italy; c Plant Health Protection Service, Emilia Romagna, Bologna, Italy; d Department of Environmental Science, Policy and Management, University of California, Berkeley, California, USA

## Abstract

The draft genome sequence of *Xylella fastidiosa* CO33 isolate, retrieved from symptomatic leaves of coffee plant intercepted in northern Italy, is reported. The CO33 genome size is 2,681,926 bp with a GC content of 51.7%.

## GENOME ANNOUNCEMENT

*Xylella fastidiosa* is a bacterium that colonizes the xylem vessels of host plants and the mouthparts of its insect vectors. This bacterium causes plant diseases of economic importance in a wide range of host plants (1). Seven complete and 12 draft *X. fastidiosa* genomes sequences are available (http://www.ncbi.nlm.nih.gov/genome/genomes/173?). In 2013, *X. fastidiosa* was detected in southern Italy, representing the first case of this quarantine bacterium becoming established in Europe (2). In July 2015, *X. fastidiosa* was also detected in Corsica, France. Due to its threat to European agriculture and the environment, the European Union (EU) strengthened customs control measures to limit importation of infected plant material. As a result several *X. fastidiosa*-infected coffee plants (*Coffea arabica*) originating from Central America were intercepted, mainly in the Netherlands (3). *X. fastidiosa* strains belonging to *X. fastidiosa* subsp. *pauca* and subsp. *fastidiosa* have been reported to infect this host (4–6). Therefore, importation of coffee plants from the Americas represents a potential reservoir of genetic and biological *X. fastidiosa* diversity.

Preliminary studies on the *X. fastidiosa* isolates recovered from these interceptions demonstrated that they belong to genetically distinct clades (7, 8). Isolate CO33, cultured from a coffee plant intercepted in northern Italy, represents a novel multilocus sequence typing profile, ST72 (G. Loconsole, personal communication). Isolates genetically related to CO33 were found in several coffee plants imported in October 2014 from Costa Rica through the Netherlands (European Food Safety Authority [EFSA] 2015). CO33 was cultured on BCYE medium and genomic DNA extracted using a commercial kit. A DNA library, paired-end sequenced with Illumina, resulted in 10,125,956 reads, representing 310-fold coverage of the expected *X. fastidiosa* genome. Reads were assembled *de novo* by EDENA, Velvet, and SOAPdenovo (9–11) with different k-mers. The best contig assemblies from each program were merged using CISA (12) and scaffolded with SSPACE (13) on the Orione instance of Galaxy (14). A final assembly of 96 scaffolds with sizes ranging from 204 to 406,234 bp and an average scaffold size of 27,936 bp was obtained. Scaffolds less than 5 kb in size were also kept if found to include data homologous to *X. fastidiosa* species by BLASTN analysis.

The draft genome of *X. fastidiosa* isolate CO33 consisted of 2,681,926 bp with a GC content of 51.7%, in agreement with other sequenced isolates that have genome sizes ranging from 2.39 to 2.73 Mbp (15). The genome sequence was annotated through the NCBI Prokaryotic Genome Automatic Annotation Pipeline (PGAAP), which identified 11 rRNA genes, 49 tRNA loci, 2,436 genes, 2,301 protein-encoding genes, 1 noncoding RNA (ncRNA), and 79 repeat regions. Alignments of reads and BLASTN searching of contigs versus available genomes of *X. fastidiosa* revealed that CO33 is genetically related to isolates belonging to different subspecies of *X. fastidiosa*. Specifically, the highest number of CO33 reads mapped either with isolates of subsp. *sandyi* (isolate Ann-1). or of subsp. *morus* (isolate MUL0034), corroborating the genetic complexity of this plant pathogen bacterium and the role of homologous recombination on *X. fastidiosa* diversity.

### Nucleotide sequence accession numbers.

This whole-genome shotgun project has been deposited at DDBJ/EMBL/GenBank under the accession number LJZW00000000. The version described in this paper is version LJZW00000000.1.
